# Local recurrence and metastasis in patients with malignant melanomas after surgery: A single-center analysis of 202 patients in South Korea

**DOI:** 10.1371/journal.pone.0213475

**Published:** 2019-03-07

**Authors:** Soo Ick Cho, Jaewon Lee, Gwanghyun Jo, Sang Wha Kim, Kyung Won Minn, Ki Yong Hong, Seong Jin Jo, Kwang Hyun Cho, Byung Jun Kim, Je-Ho Mun

**Affiliations:** 1 Department of Dermatology, Seoul National University College of Medicine, Seoul, South Korea; 2 Department of Plastic and Reconstructive Surgery, Seoul National University College of Medicine, Seoul, South Korea; 3 Department of Plastic and Reconstructive Surgery, Dongguk University Ilsan Hospital, Goyang, South Korea; University of South Alabama Mitchell Cancer Institute, UNITED STATES

## Abstract

Malignant melanoma (MM) is a lethal skin cancer in Western countries. Although the incidence is low in Asians compared to that in Caucasians, it is increasing. However, literature regarding risk factors for prognosis of MM patients who have undergone surgical excision in Asian is limited. This study aimed to investigate the predictive factors for local recurrence and metastasis in MM patients who underwent surgical treatment at a single tertiary-level hospital in Korea. Patients who underwent surgery for MM at our institution between January 1998 and December 2014 were analyzed. We retrospectively investigated risk factors for local recurrence and metastasis after surgery. In cases with distant metastasis, tumor thickness (adjusted Hazard Ratio (HR), 6.139; 95% confidence interval (CI), 2.152 to 17.509; *P* = 0.001) and increased mitotic number [(0-1/mm^2^ vs 2-6/mm^2^: adjusted HR, 4.483; 95% CI, 1.233 to 16.303; *P* = 0.023); (0-1/mm^2^ vs > 6/mm^2^: adjusted HR, 10.316; 95% CI, 2.871 to 37.063; *P* < 0.001)] were associated with risk in multivariate analysis. Regarding local recurrence, tumor thickness (T4 [≥4mm] vs T1) was found to be a significant risk factor (adjusted HR, 8.461; 95% CI, 2.514 to 28.474; *P* = 0.001). Our data revealed tumor thickness and increased mitotic count were significant risk factors for local recurrence and distant metastasis in Korean patients with MM after surgery.

## Introduction

Malignant melanoma (MM) is a fatal skin tumor originating from melanocytes. Although its reported age-standardized incidence is only 0.4–0.6 per 100,000 people in Korea, it is one of the leading causes of death among skin cancers. [1] Biological and environmental factors such as the number of common or atypical nevi, intermittent and intense sun exposure, and some phenotypic characteristics (light, fair color of skin, hair and eyes; family history of MM) increase the risk of MM.[2–4] Various epidemiologic and clinical features of MM have been studied to date.[5–7]

The incidence of MM is lower in eastern countries than in western countries.[8] Recently, however, the incidence and prevalence of MM has been increasing annually.[1] MM in Asian patients affects different sites and exhibits different clinical features and worse prognosis than those in western patients.[9–11]

To our knowledge, there have been few studies regarding the risk factors for local recurrence and metastasis in MM in Korea.[12–14] The aim of this study was to report the surgical experience of patients with MM at a tertiary hospital in South Korea over the past 17 years. We investigated key predictive factors associated with local recurrence and metastasis after surgery.

## Materials and methods

### Study design

We conducted a retrospective analysis of subjects who underwent curative surgery for MM at the Seoul National University Hospital between January 1998 and December 2014. Demographic, clinical, and follow-up data of subjects were obtained from electronic medical records. Histopathological information was obtained from pathology reports from the Department of Pathology.

The following information was obtained for each subject: (1) demographic data (sex, age); (2) history of local recurrence or metastasis; and (3) clinical and histopathological features (tumor site, ulceration, resection margin, tumor thickness, information about lymph nodes, mitotic counts, and histologic subtype). Local recurrence and metastasis were identified at outpatient clinic visits.

### Statistical analysis

IBM SPSS statistics version 23.0 (IBM corp., Armonk, NY, USA) was used for statistical analysis. The differences in local recurrence or metastasis associated with demographic factors (sex and age), clinical and histopathological data (tumor site, ulceration, resection margin, tumor thickness, information about lymph nodes, mitotic counts, and histologic subtype) were considered statistically significant if the *P*-value was < 0.05. Univariate and multivariate Cox regression models for local recurrence and metastasis were analyzed with the forward likelihood ratio method (*P*-value < 0.05 was to be included in the analysis).

### Ethics statement

The study protocol was approved by the Institutional Review Board of Seoul National University Hospital (IRB No. 1407-080-594). Informed consent was waived as the subjects were de-identified.

## Results

### Demographic and clinical characteristics of the study population

A total of 202 patients were enrolled in this study, including 85 (42.08%) men and 117 (57.92%) women. The mean age at diagnosis was 58.05 ± 12.60 years (mean ± standard deviation [S.D.], range 23–83 years). The acral site was the most common tumor site (134, 66.34%), followed by the upper and lower extremities (26, 12.87%), trunk (22, 10.89%), and head and neck (20, 9.90%). The most common histologic subtype of MM was acral lentiginous melanoma (115, 56.93%), followed by nodular (42, 20.79%), superficial spreading (29, 14.36%), lentigo maligna (14, 6.93%), and desmoplastic (2, 0.99%) lesions. Most of the surgical resection margin was free from tumor (187, 96.39%). Lymph node (LN) exploration was performed in 79 patients (39.11%). Among all the patients who underwent LN exploration, 19 patients were positive (24.05%). All demographic and clinical variables of patients are summarized in Table 1.

**Table 1:** Demographic and clinical factors of subjects.

	Number of patients (%)
Sex	
Male	85 (42.08%)
Female	117 (57.92%)
Age at diagnosis	
< 64	138 (68.32%)
≥ 65	64 (31.68%)
Body site	
Acral	134 (66.34%)
Head/neck	20 (9.90%)
Upper/lower extremity	26 (12.87%)
Trunk	22 (10.89%)
Ulceration	
No	122 (60.40%)
Yes	80 (39.60%)
Resection margin positive	
No	187 (96.39%)
Yes	7 (3.61%)
not assessed	8
Breslow thickness	
T1	93 (46.04%)
T2	33 (16.34%)
T3	29 (14.35%)
T4	47 (23.27%)
Lymph node exploration	
Not done	123 (60.89%)
Done, negative	60 (29.70%)
Done, positive	19 (9.41%)
Mitosis	
0-1/mm^2^	52 (37.14%)
2-6/mm^2^	47 (33.57%)
> 6/mm^2^	41 (29.29%)
not assessed	62
Histologic subtype	
Acral lentiginous	115 (56.93%)
Superficial spreading	29 (14.36%)
Lentigo maligna	14 (6.93%)
Nodular	42 (20.79%)
Desmoplastic	2 (0.99%)

### Local recurrence and association with clinicopathological factors

During a mean follow-up period (recurrence or metastasis) of 40.54 ± 35.16 months (mean ± S.D., range 1–201 months), 19 patients (9.41%) developed local recurrence after surgery. In univariate analysis, tumors with a positive resection margin, increased tumor thickness (T4 [≥ 4mm] vs T1), positive LN after LN exploration, tumor with increased mitotic number, and histologic subtypes (nodular vs acral lentiginous) were significantly associated with higher rate of local recurrence. In a multivariate model, tumor thickness (T4 vs T1) (adjusted Hazard Ratio (HR), 8.461; 95% confidence interval (CI), 2.514 to 28.474; *P* = 0.001) was associated with an increased risk of local recurrence (Table 2).

**Table 2:** An association of clinical and histopathologic variables and local recurrence of malignant melanoma.

	Univariate model		Multivariate model (Best Model)	
	Hazard ratio (95% CI)	*P*-value	Hazard ratio (95% CI)	*P*-value
Sex				
Male	Reference			
Female	1.106 (0.433–2.824)	0.833		
Age at diagnosis	1.041 (0.999–1.089)	0.056		
< 64	Reference			
≥ 65	2.460 (0.998–6.063)	0.050		
Body site		0.239		
Acral	Reference			
Head/neck	2.257 (0.712–7.159)	0.167		
Upper/lower extremity	0.585 (0.073–4.387)	0.585		
Trunk	2.714 (0.754–9.775)	0.127		
Ulceration				
No	Reference			
Yes	2.142 (0.866–5.301)	0.099		
Resection margin positive				
No	Reference			
Yes	4.640 (1.331–16.179)	0.016		
Breslow thickness				
T1	Reference		Reference	
T2	0.590 (0.069–5.062)	0.631	0.723 (0.081–6.483)	0.772
T3	0.000 (0.000)	0.987	0.000 (0.000)	0.983
T4	10.788 (3.820–30.467)	< 0.001	8.461 (2.514–28.474)	0.001
Lymph node exploration				
Not done	Reference			
Done, negative	1.395 (0.467–4.166)	0.551		
Done, positive	9.531 (2.666–34.067)	0.001		
Mitosis				
0-1/mm^2^	Reference			
2-6/mm^2^	4.714 (0.490–45.366)	0.180		
> 6/mm^2^	24.347 (3.070–193.083)	0.003		
Histologic subtype				
Acral lentiginous	Reference			
Superficial spreading	1.406 (0.283–6.988)	0.677		
Lentigo maligna	2.234 (0.448–11.144)	0.327		
Nodular	9.698 (3.304–28.473)	< 0.001		
Desmoplastic	6.518 (0.746–56.928)	0.090		

### Distant metastasis and association of clinicopathological factors

During the follow-up period, 46 patients (22.77%) developed distant metastasis after surgery. Univariate analysis showed that body site (trunk), ulceration, increased tumor thickness (T3/T4 vs T1), positive LN, increased mitotic number, and histologic subtypes (nodular) were significantly associated with a higher rate of metastasis. Results of a multivariate model analysis showed that tumor thickness (T4 vs T1) (adjusted HR, 6.139; 95% CI, 2.152 to 17.509; *P* = 0.001) and increased mitotic number [(0-1/mm^2^ vs 2-6/mm^2^: adjusted HR, 4.483; 95% CI, 1.233 to 16.303; *P* = 0.023); (0-1/mm^2^ vs > 6/mm^2^: adjusted HR, 10.316; 95% CI, 2.871 to 37.063; *P* < 0.001)] were associated with an increased risk of distant metastasis (Table 3).

**Table 3:** An association of clinical and histopathologic variables and distant metastasis of malignant melanoma.

	Univariate model		Multivariate model (Best Model)	
	Hazard ratio (95% CI)	*P*-value	Hazard ratio (95% CI)	*P*-value
Sex				
Male	Reference			
Female	0.793 (0.442–1.426)	0.439		
Age at diagnosis	1.003 (0.979–1.028)	0.803		
< 64	Reference			
≥ 65	0.904 (0.467–1.746)	0.763		
Body site				
Acral	Reference			
Head/neck	0.509 (0.145–2.601)	0.509		
Upper/lower extremity	1.708 (0.769–3.794)	0.188		
Trunk	3.042 (1.494–6.193)	0.002		
Ulceration				
No	Reference			
Yes	1.786 (1.000–3.190)	0.050		
Resection margin positive				
No	Reference			
Yes	0.762 (0.105–5.549)	0.788		
Breslow thickness				
T1	Reference		Reference	
T2	1.575 (0.515–4.816)	0.426	0.971 (0.255–3.704)	0.966
T3	7.571 (3.160–18.138)	< 0.001	2.254 (0.698–7.274)	0.174
T4	10.337 (4.493–23.785)	< 0.001	6.139 (2.152–17.509)	0.001
Lymph node exploration				
Not done	Reference			
Done, negative	0.803 (0.364–1.774)	0.588		
Done, positive	4.897 (2.144–11.185)	< 0.001		
Mitosis				
0-1/mm^2^	Reference		Reference	
2-6/mm^2^	6.126 (1.742–21.537)	0.005	4.483 (1.233–16.303)	0.023
> 6/mm^2^	13.438 (3.927–45.987)	< 0.001	10.316 (2.871–37.063)	< 0.001
Histologic subtype				
Acral lentiginous	Reference			
Superficial spreading	1.226 (0.488–3.077)	0.665		
Lentigo maligna	0.470 (0.063–3.520)	0.462		
Nodular	6.197 (3.255–11.797)	< 0.001		
Desmoplastic	0.000 (0.000)	0.986		

## Discussion

There have been reports comparing surgical outcomes and prognostic factors in MM to date.[15, 16] Thomas et al.[17] reported an association between demographic and clinical factors with locoregional recurrence and death in 900 MM patients after surgery. According to the study, male sex, tumors with greater thickness, and tumors with ulceration were associated with increased local recurrence and death. Generally, acral lentiginous melanoma is considered to have worse prognosis than other subtypes because it is often diagnosed later than MM located on the trunk or limbs.[18–20] Kato et al.[19] compared the prognosis of patients from the first half of the study period (1969–1982) with those from the second half (1983–1996) and reported an improving trend in prognosis. The authors mentioned that increased educational efforts regarding early detection of acral melanoma might have some contribution to lowering the risk of the disease. A recent report revealed that acral lentiginous melanoma had no significant prognostic difference than non-acral lentiginous melanoma.[21] The results of our study showed similar results. MM on the acral area did not have a higher incidence of local recurrence or distant metastasis than MM located on other anatomical sites.

In our study, ulceration was identified in 39.60% of patients, which was similar to the ulceration status reported in a previous study (36.60%) by Thomas et al.[17] We found a statistically significant difference for ulceration status with regard to poor prognosis on univariate analysis; however, there was no significant statistical effect after multivariate analysis. Only tumor thickness remained a significant factor. Many studies have shown an association between ulceration in MM and poor prognosis.[21–23] Conversely, other studies have found there was no association.[24, 25] There are divergent results in the published Korean literature.[26, 27] Therefore, further studies are necessary to determine the impact of ulceration status on the prognosis of MM.

The distribution of demographic and clinical characteristics of subjects in this study including sex, histological subtypes, or tumor thickness was similar with that of reported studies from Korea or from other Asian countries.[25, 28, 29] Roh et al.[25] reported the prognostic outcomes of acral melanoma patients and indicated that tumor thickness and advanced clinical stage were associated with a worse prognosis. However, they were unable to find any association between sex, tumor sites, or histological subtypes and worse prognosis. Our findings in this study are in line with that of Roh et al.[25] and with other studies from Japan and Western countries.[19, 30, 31]

Our study has some limitations. First, this is a single-centered, retrospective study. Second, the results of our study do not represent the general prognosis of MM because we enrolled patients who underwent surgery. Third, recent studies reported that gene expression signature in MM might have a role in prognosis, although this study did not include genomic data. [32–35] Despite these limitations, we report the surgical experience with one of the largest numbers of MM patients in Korea. Therefore, the results of our study may provide valuable information for clinicians and patients, especially, when counseling MM patients who have impending surgery in Asia.

In conclusion, our study showed tumor thickness was associated with local recurrence of MM after surgery. Regarding distant metastasis, tumor thickness and increased mitotic number were significant factors.

## Supporting information

S1 Dataset Demographic, clinical, pathologic factors and outcome of patients with malignant melanoma (N = 202). (DOCX)
