# Supplementary material for: Local recurrence and metastasis in patients with malignant melanomas after surgery: A single-center analysis of 202 patients in South Korea
Source: PLoS One. 2019 Mar 7;14(3):e0213475. doi: 10.1371/journal.pone.0213475 (PMC6405088; doi:10.1371/journal.pone.0213475)
Supplement: S1 Dataset — (DOCX) [file pone.0213475.s001.docx]

**S1 Dataset**. Demographic, clinical, pathologic factors and outcome of patients with malignant melanoma (N=202)

|  | Sex | Age at diagnosis | Body site | Ulceration | Resection margin positive | Breslow thickness | Lymph node exploration | Mitosis | Histologic subtype | Follow up period (months) | Local recurrence | Distant metastasis | Disease Free interval (months) |
| --- | --- | --- | --- | --- | --- | --- | --- | --- | --- | --- | --- | --- | --- |
| 1 | F | 68 | Acral | No | No | T1 | Not done | NA | Acral lentiginous | 201 | No | No | 201 |
| 2 | F | 73 | Head/neck | Yes | No | T1 | Not done | 1 | Lentigo maligna | 97 | Yes | No | 37 |
| 3 | M | 66 | Acral | No | No | T4 | Done, negative | NA | Acral lentiginous | 66 | No | No | 66 |
| 4 | F | 51 | Trunk | No | No | T1 | Not done | NA | Superficial spreading | 128 | No | Yes | 60 |
| 5 | M | 73 | Acral | No | No | T1 | Not done | NA | Acral lentiginous | 48 | No | No | 48 |
| 6 | F | 61 | Acral | No | No | T3 | Not done | 3 | Acral lentiginous | 87 | No | Yes | 72 |
| 7 | M | 71 | Acral | No | No | T1 | Not done | 3 | Acral lentiginous | 59 | No | No | 59 |
| 8 | F | 56 | Head/neck | No | No | T1 | Not done | 4 | Lentigo maligna | 146 | Yes | No | 34 |
| 9 | F | 73 | Acral | No | No | T1 | Not done | NA | Acral lentiginous | 64 | No | No | 64 |
| 10 | M | 76 | Acral | Yes | No | T4 | Not done | NA | Nodular | 112 | Yes | No | 50 |
| 11 | M | 77 | Acral | Yes | No | T2 | Done, negative | 16 | Acral lentiginous | 30 | No | No | 30 |
| 12 | M | 68 | Acral | Yes | No | T4 | Done, negative | 2 | Acral lentiginous | 22 | No | Yes | 15 |
| 13 | M | 78 | Acral | Yes | No | T2 | Not done | 0 | Acral lentiginous | 41 | No | Yes | 31 |
| 14 | F | 56 | Acral | Yes | No | T1 | Not done | 10 | Acral lentiginous | 61 | No | Yes | 35 |
| 15 | F | 83 | Acral | Yes | No | T2 | Not done | 5 | Acral lentiginous | 49 | No | No | 49 |
| 16 | F | 61 | Trunk | No | No | T1 | Not done | NA | Superficial spreading | 64 | No | No | 64 |
| 17 | F | 57 | Acral | No | No | T1 | Done, negative | 6 | Nodular | 19 | No | No | 19 |
| 18 | M | 74 | Head/neck | Yes | No | T2 | Not done | 1 | Lentigo maligna | 56 | No | No | 56 |
| 19 | M | 78 | Acral | Yes | No | T2 | Done, negative | 3 | Acral lentiginous | 16 | No | Yes | 8 |
| 20 | F | 57 | Acral | Yes | No | T1 | Done, negative | 1 | Acral lentiginous | 34 | No | No | 34 |
| 21 | F | 58 | Acral | Yes | No | T3 | Not done | NA | Acral lentiginous | 11 | No | Yes | 7 |
| 22 | F | 50 | Upper/lower extremity | No | No | T1 | Done, negative | 1 | Superficial spreading | 12 | No | No | 12 |
| 23 | F | 61 | Upper/lower extremity | No | No | T4 | Not done | NA | Superficial spreading | 94 | Yes | No | 16 |
| 24 | M | 51 | Acral | No | No | T1 | Not done | 0 | Lentigo maligna | 48 | No | No | 48 |
| 25 | M | 56 | Acral | Yes | No | T1 | Not done | NA | Acral lentiginous | 76 | No | No | 76 |
| 26 | F | 32 | Upper/lower extremity | Yes | No | T2 | Not done | NA | Superficial spreading | 96 | No | Yes | 79 |
| 27 | F | 51 | Trunk | No | No | T3 | Not done | NA | Superficial spreading | 30 | No | Yes | 27 |
| 28 | F | 79 | Acral | Yes | No | T1 | Not done | 13 | Nodular | 56 | No | Yes | 6 |
| 29 | F | 64 | Acral | No | No | T1 | Done, negative | NA | Acral lentiginous | 149 | No | No | 149 |
| 30 | F | 60 | Acral | Yes | No | T4 | Not done | 7 | Acral lentiginous | 27 | No | Yes | 9 |
| 31 | F | 64 | Acral | Yes | No | T4 | Not done | 0 | Nodular | 81 | No | No | 81 |
| 32 | M | 62 | Upper/lower extremity | No | No | T3 | Done, negative | 1 | Nodular | 25 | No | No | 25 |
| 33 | F | 55 | Trunk | No | No | T2 | Not done | NA | Superficial spreading | 164 | No | No | 164 |
| 34 | M | 61 | Trunk | No | No | T4 | Not done | NA | Nodular | 35 | No | Yes | 8 |
| 35 | F | 74 | Acral | No | No | T1 | Done, negative | 1 | Superficial spreading | 26 | No | No | 26 |
| 36 | F | 69 | Acral | No | No | T1 | Not done | 1 | Acral lentiginous | 60 | No | No | 60 |
| 37 | F | 23 | Trunk | No | No | T1 | Not done | NA | Superficial spreading | 61 | No | No | 61 |
| 38 | F | 70 | Head/neck | Yes | No | T2 | Not done | NA | Superficial spreading | 19 | No | No | 19 |
| 39 | M | 64 | Acral | Yes | No | T4 | Done, negative | 15 | Nodular | 11 | No | No | 11 |
| 40 | F | 71 | Head/neck | No | No | T2 | Not done | NA | Lentigo maligna | 56 | No | No | 56 |
| 41 | F | 59 | Head/neck | Yes | No | T1 | Not done | NA | Lentigo maligna | 33 | No | No | 33 |
| 42 | M | 49 | Head/neck | No | No | T4 | Done, negative | NA | Superficial spreading | 58 | No | No | 58 |
| 43 | F | 56 | Acral | Yes | Yes | T1 | Not done | NA | Acral lentiginous | 60 | No | Yes | 51 |
| 44 | F | 57 | Acral | Yes | No | T3 | Done, negative | NA | Acral lentiginous | 45 | No | Yes | 23 |
| 45 | F | 66 | Acral | No | Yes | T2 | Not done | 0 | Acral lentiginous | 84 | No | No | 84 |
| 46 | F | 57 | Acral | No | No | T2 | Not done | NA | Acral lentiginous | 64 | No | No | 64 |
| 47 | M | 62 | Acral | No | No | T3 | Done, negative | 14 | Nodular | 8 | No | No | 8 |
| 48 | M | 61 | Acral | No | No | T1 | Not done | NA | Acral lentiginous | 70 | No | No | 70 |
| 49 | F | 58 | Head/neck | No | No | T2 | Not done | NA | Lentigo maligna | 60 | No | No | 60 |
| 50 | F | 50 | Acral | No | Yes | T3 | Not done | 1 | Acral lentiginous | 16 | No | No | 16 |
| 51 | M | 76 | Trunk | No | No | T4 | Not done | NA | Superficial spreading | 1 | No | No | 1 |
| 52 | M | 41 | Acral | No | No | T3 | Not done | NA | Acral lentiginous | 6 | No | No | 6 |
| 53 | F | 56 | Acral | No | Yes | T1 | Not done | NA | Acral lentiginous | 81 | Yes | No | 19 |
| 54 | F | 63 | Acral | Yes | No | T4 | Not done | NA | Acral lentiginous | 100 | Yes | No | 12 |
| 55 | M | 74 | Trunk | No | No | T4 | Not done | NA | Nodular | 20 | No | Yes | 9 |
| 56 | M | 79 | Head/neck | No | No | T4 | Done, positive | 2 | Nodular | 6 | No | No | 6 |
| 57 | F | 45 | Acral | Yes | No | T1 | Done, negative | NA | Acral lentiginous | 145 | No | No | 145 |
| 58 | M | 62 | Acral | Yes | No | T1 | Not done | NA | Acral lentiginous | 35 | No | No | 35 |
| 59 | M | 52 | Acral | Yes | No | T4 | Not done | NA | Acral lentiginous | 21 | No | No | 21 |
| 60 | F | 68 | Upper/lower extremity | No | No | T4 | Not done | 2 | Nodular | 17 | No | Yes | 12 |
| 61 | M | 36 | Acral | Yes | No | T2 | Not done | 2 | Acral lentiginous | 50 | No | No | 50 |
| 62 | F | 75 | Head/neck | No | No | T1 | Not done | NA | Lentigo maligna | 1 | No | No | 1 |
| 63 | F | 57 | Acral | No | No | T2 | Not done | NA | Acral lentiginous | 23 | No | No | 23 |
| 64 | F | 49 | Upper/lower extremity | No | No | T1 | Not done | NA | Superficial spreading | 136 | No | No | 136 |
| 65 | M | 63 | Acral | No | No | T1 | Not done | NA | Acral lentiginous | 120 | No | No | 120 |
| 66 | F | 67 | Acral | Yes | No | T2 | Not done | NA | Acral lentiginous | 57 | No | No | 57 |
| 67 | F | 66 | Trunk | No | No | T4 | Done, positive | 9 | Nodular | 10 | Yes | Yes | 8 |
| 68 | F | 67 | Acral | No | No | T1 | Not done | NA | Acral lentiginous | 123 | No | No | 123 |
| 69 | M | 49 | Acral | No | No | T1 | Not done | NA | Acral lentiginous | 83 | No | No | 83 |
| 70 | F | 54 | Head/neck | No | No | T4 | Not done | NA | Lentigo maligna | 58 | No | No | 58 |
| 71 | M | 65 | Acral | Yes | NA | T3 | Not done | NA | Acral lentiginous | 79 | No | Yes | 78 |
| 72 | F | 34 | Upper/lower extremity | No | No | T2 | Not done | NA | Nodular | 112 | No | No | 112 |
| 73 | F | 44 | Upper/lower extremity | No | NA | T4 | Not done | NA | Nodular | 18 | No | Yes | 3 |
| 74 | M | 57 | Upper/lower extremity | No | No | T1 | Not done | 4 | Superficial spreading | 19 | No | No | 19 |
| 75 | F | 47 | Acral | No | NA | T1 | Done, negative | NA | Acral lentiginous | 90 | No | No | 26 |
| 76 | F | 69 | Acral | No | No | T2 | Not done | NA | Acral lentiginous | 55 | No | No | 55 |
| 77 | M | 47 | Acral | No | NA | T4 | Done, negative | NA | Acral lentiginous | 113 | Yes | No | 58 |
| 78 | F | 43 | Acral | No | No | T3 | Not done | NA | Acral lentiginous | 23 | No | Yes | 19 |
| 79 | M | 75 | Acral | Yes | Yes | T4 | Not done | 2 | Desmoplastic | 110 | Yes | No | 88 |
| 80 | F | 78 | Acral | Yes | No | T4 | Done, positive | 16 | Nodular | 2 | No | No | 2 |
| 81 | F | 47 | Acral | Yes | No | T1 | Not done | NA | Acral lentiginous | 23 | No | No | 23 |
| 82 | M | 62 | Acral | No | No | T1 | Not done | 0 | Acral lentiginous | 40 | No | No | 40 |
| 83 | F | 57 | Acral | Yes | NA | T1 | Not done | NA | Acral lentiginous | 96 | No | No | 96 |
| 84 | F | 34 | Acral | No | No | T1 | Not done | NA | Acral lentiginous | 93 | No | No | 93 |
| 85 | M | 72 | Acral | Yes | No | T4 | Done, negative | 35 | Acral lentiginous | 8 | No | No | 8 |
| 86 | F | 39 | Trunk | No | No | T4 | Not done | 6 | Nodular | 100 | No | Yes | 48 |
| 87 | F | 49 | Acral | Yes | No | T1 | Not done | 0 | Acral lentiginous | 32 | No | No | 32 |
| 88 | M | 53 | Acral | Yes | No | T1 | Not done | 5 | Acral lentiginous | 82 | No | Yes | 51 |
| 89 | M | 62 | Acral | No | NA | T3 | Done, positive | 10 | Acral lentiginous | 26 | No | Yes | 3 |
| 90 | F | 65 | Acral | No | No | T1 | Done, negative | 1 | Acral lentiginous | 40 | No | No | 40 |
| 91 | M | 50 | Head/neck | No | No | T1 | Not done | 0 | Lentigo maligna | 59 | No | No | 59 |
| 92 | M | 56 | Acral | Yes | No | T2 | Not done | 9 | Acral lentiginous | 51 | No | No | 51 |
| 93 | M | 75 | Acral | Yes | No | T2 | Done, negative | 8 | Nodular | 6 | No | No | 6 |
| 94 | M | 60 | Trunk | No | No | T2 | Not done | 0 | Superficial spreading | 60 | No | No | 60 |
| 95 | M | 67 | Head/neck | No | No | T3 | Not done | 10 | Superficial spreading | 80 | No | No | 80 |
| 96 | F | 47 | Acral | No | No | T1 | Not done | NA | Acral lentiginous | 23 | No | No | 23 |
| 97 | M | 61 | Acral | No | No | T1 | Not done | 2 | Acral lentiginous | 10 | No | No | 10 |
| 98 | F | 58 | Acral | Yes | No | T1 | Not done | 0 | Acral lentiginous | 57 | No | No | 57 |
| 99 | M | 46 | Upper/lower extremity | No | No | T4 | Not done | 30 | Nodular | 29 | No | Yes | 25 |
| 100 | F | 69 | Acral | Yes | No | T1 | Not done | 0 | Acral lentiginous | 52 | No | No | 52 |
| 101 | M | 44 | Upper/lower extremity | Yes | No | T2 | Not done | 3 | Lentigo maligna | 59 | No | No | 59 |
| 102 | M | 76 | Acral | Yes | No | T3 | Not done | 18 | Acral lentiginous | 47 | No | No | 47 |
| 103 | F | 50 | Trunk | No | No | T4 | Not done | 5 | Superficial spreading | 74 | No | Yes | 16 |
| 104 | M | 45 | Upper/lower extremity | No | No | T3 | Not done | 5 | Nodular | 38 | No | Yes | 16 |
| 105 | F | 57 | Acral | Yes | No | T3 | Not done | 3 | Acral lentiginous | 44 | No | No | 44 |
| 106 | F | 49 | Head/neck | No | No | T1 | Not done | 0 | Lentigo maligna | 12 | No | No | 12 |
| 107 | F | 57 | Acral | No | No | T1 | Done, negative | 0 | Acral lentiginous | 60 | No | No | 60 |
| 108 | F | 52 | Upper/lower extremity | No | NA | T1 | Not done | NA | Superficial spreading | 67 | No | No | 67 |
| 109 | F | 82 | Acral | Yes | No | T2 | Not done | 21 | Acral lentiginous | 11 | No | No | 11 |
| 110 | F | 71 | Acral | No | No | T1 | Done, negative | 1 | Acral lentiginous | 29 | No | No | 29 |
| 111 | F | 65 | Acral | No | No | T1 | Not done | NA | Acral lentiginous | 56 | No | No | 56 |
| 112 | F | 51 | Acral | No | No | T1 | Done, negative | 2 | Acral lentiginous | 24 | No | No | 24 |
| 113 | M | 59 | Head/neck | Yes | No | T3 | Not done | 17 | Nodular | 13 | No | Yes | 4 |
| 114 | F | 51 | Acral | Yes | No | T1 | Not done | NA | Acral lentiginous | 48 | No | No | 48 |
| 115 | M | 64 | Acral | Yes | No | T3 | Not done | 5 | Acral lentiginous | 50 | No | No | 50 |
| 116 | F | 33 | Head/neck | Yes | No | T1 | Not done | NA | Superficial spreading | 49 | No | No | 49 |
| 117 | M | 42 | Upper/lower extremity | No | No | T3 | Done, positive | 22 | Nodular | 15 | No | No | 15 |
| 118 | M | 52 | Trunk | Yes | No | T4 | Not done | 16 | Nodular | 10 | No | Yes | 5 |
| 119 | M | 71 | Upper/lower extremity | Yes | No | T1 | Not done | 10 | Acral lentiginous | 55 | No | No | 55 |
| 120 | F | 35 | Acral | Yes | No | T2 | Not done | 6 | Acral lentiginous | 44 | No | No | 44 |
| 121 | M | 53 | Acral | Yes | No | T3 | Not done | 6 | Nodular | 26 | No | Yes | 11 |
| 122 | M | 69 | Acral | No | No | T1 | Not done | NA | Acral lentiginous | 31 | No | No | 31 |
| 123 | F | 27 | Upper/lower extremity | No | No | T1 | Not done | NA | Superficial spreading | 25 | No | Yes | 7 |
| 124 | F | 58 | Acral | No | No | T2 | Not done | 7 | Acral lentiginous | 47 | No | Yes | 29 |
| 125 | M | 37 | Acral | Yes | No | T1 | Not done | 0 | Acral lentiginous | 42 | No | No | 42 |
| 126 | M | 70 | Acral | Yes | No | T2 | Not done | 12 | Acral lentiginous | 48 | No | Yes | 36 |
| 127 | F | 63 | Trunk | Yes | No | T4 | Done, negative | 21 | Nodular | 41 | Yes | No | 3 |
| 128 | F | 49 | Acral | No | No | T2 | Not done | 15 | Acral lentiginous | 24 | No | No | 24 |
| 129 | F | 54 | Acral | No | No | T1 | Not done | 0 | Acral lentiginous | 41 | No | No | 41 |
| 130 | F | 53 | Acral | No | No | T1 | Done, negative | 0 | Acral lentiginous | 38 | No | No | 38 |
| 131 | M | 58 | Acral | No | No | T1 | Not done | 1 | Acral lentiginous | 12 | No | No | 12 |
| 132 | M | 30 | Acral | No | No | T4 | Done, negative | 1 | Acral lentiginous | 38 | No | No | 38 |
| 133 | F | 52 | Acral | No | No | T1 | Done, negative | 10 | Acral lentiginous | 39 | No | No | 39 |
| 134 | M | 54 | Trunk | No | No | T1 | Done, negative | 0 | Superficial spreading | 41 | No | No | 41 |
| 135 | F | 59 | Acral | No | No | T3 | Not done | 0 | Acral lentiginous | 33 | No | No | 33 |
| 136 | F | 54 | Acral | No | No | T1 | Not done | 1 | Acral lentiginous | 30 | No | No | 30 |
| 137 | M | 53 | Acral | No | No | T1 | Done, negative | 2 | Acral lentiginous | 35 | No | No | 35 |
| 138 | F | 49 | Upper/lower extremity | No | No | T1 | Not done | 0 | Acral lentiginous | 27 | No | No | 27 |
| 139 | F | 74 | Acral | No | No | T1 | Done, negative | 0 | Acral lentiginous | 30 | No | No | 30 |
| 140 | F | 52 | Upper/lower extremity | No | No | T4 | Done, negative | 1 | Nodular | 32 | No | No | 32 |
| 141 | F | 64 | Acral | Yes | No | T1 | Not done | 2 | Acral lentiginous | 34 | No | No | 34 |
| 142 | F | 72 | Trunk | No | No | T4 | Done, positive | 7 | Nodular | 38 | Yes | No | 8 |
| 143 | F | 77 | Acral | Yes | No | T4 | Done, negative | 3 | Acral lentiginous | 1 | No | No | 1 |
| 144 | M | 65 | Upper/lower extremity | No | No | T4 | Done, positive | 2 | Nodular | 17 | No | No | 17 |
| 145 | F | 75 | Acral | No | No | T1 | Not done | 4 | Acral lentiginous | 36 | Yes | No | 33 |
| 146 | F | 36 | Acral | Yes | No | T1 | Not done | 1 | Acral lentiginous | 28 | No | No | 28 |
| 147 | M | 73 | Acral | Yes | No | T4 | Not done | 46 | Nodular | 1 | No | No | 1 |
| 148 | F | 61 | Trunk | No | No | T4 | Done, positive | 3 | Nodular | 32 | No | Yes | 25 |
| 149 | F | 58 | Head/neck | No | No | T1 | Not done | 0 | Lentigo maligna | 29 | No | Yes | 12 |
| 150 | F | 65 | Acral | Yes | No | T4 | Done, positive | 4 | Nodular | 13 | Yes | Yes | 6 |
| 151 | M | 39 | Head/neck | Yes | No | T4 | Not done | 28 | Nodular | 24 | Yes | No | 9 |
| 152 | F | 40 | Trunk | No | No | T4 | Done, negative | 1 | Nodular | 7 | No | No | 7 |
| 153 | F | 63 | Upper/lower extremity | Yes | No | T4 | Done, positive | 18 | Nodular | 26 | No | Yes | 10 |
| 154 | M | 53 | Acral | No | No | T1 | Not done | NA | Acral lentiginous | 22 | No | No | 22 |
| 155 | F | 61 | Acral | No | No | T4 | Done, positive | 4 | Acral lentiginous | 25 | No | No | 25 |
| 156 | F | 70 | Head/neck | No | No | T1 | Not done | 0 | Lentigo maligna | 6 | No | No | 6 |
| 157 | F | 56 | Acral | No | No | T3 | Done, negative | 2 | Acral lentiginous | 24 | No | No | 24 |
| 158 | F | 62 | Acral | No | No | T3 | Done, negative | 10 | Nodular | 25 | No | Yes | 10 |
| 159 | M | 74 | Acral | Yes | No | T3 | Done, positive | 0 | Acral lentiginous | 2 | No | No | 2 |
| 160 | M | 52 | Acral | No | No | T1 | Done, negative | 0 | Acral lentiginous | 18 | No | No | 18 |
| 161 | F | 75 | Acral | Yes | No | T2 | Not done | 10 | Acral lentiginous | 12 | No | No | 12 |
| 162 | F | 64 | Upper/lower extremity | Yes | No | T4 | Done, negative | 1 | Nodular | 25 | No | Yes | 9 |
| 163 | M | 41 | Trunk | No | No | T1 | Done, negative | 5 | Superficial spreading | 21 | No | No | 21 |
| 164 | M | 57 | Acral | No | No | T1 | Not done | 1 | Acral lentiginous | 17 | No | No | 17 |
| 165 | M | 50 | Acral | No | No | T4 | Not done | 4 | Acral lentiginous | 24 | No | Yes | 16 |
| 166 | F | 45 | Acral | No | No | T1 | Done, negative | 2 | Acral lentiginous | 17 | No | No | 17 |
| 167 | M | 58 | Acral | Yes | No | T1 | Done, positive | 8 | Acral lentiginous | 14 | No | Yes | 8 |
| 168 | F | 67 | Acral | No | No | T1 | Done, negative | 1 | Acral lentiginous | 17 | No | No | 17 |
| 169 | F | 57 | Acral | No | No | T2 | Not done | 0 | Acral lentiginous | 18 | No | No | 18 |
| 170 | F | 78 | Acral | Yes | No | T4 | Done, positive | 10 | Acral lentiginous | 19 | No | No | 19 |
| 171 | F | 30 | Upper/lower extremity | No | No | T2 | Done, negative | 0 | Nodular | 18 | No | No | 18 |
| 172 | M | 62 | Acral | Yes | No | T3 | Done, negative | 0 | Acral lentiginous | 12 | No | No | 12 |
| 173 | F | 48 | Acral | No | Yes | T1 | Not done | 0 | Acral lentiginous | 17 | No | No | 17 |
| 174 | M | 56 | Acral | Yes | No | T2 | Done, positive | 2 | Acral lentiginous | 19 | No | No | 19 |
| 175 | F | 45 | Trunk | No | No | T1 | Not done | NA | Superficial spreading | 18 | No | No | 18 |
| 176 | F | 71 | Acral | Yes | No | T1 | Done, negative | 1 | Superficial spreading | 15 | No | No | 15 |
| 177 | F | 65 | Acral | Yes | No | T1 | Done, negative | 15 | Acral lentiginous | 19 | Yes | No | 19 |
| 178 | M | 76 | Acral | Yes | Yes | T4 | Done, positive | 22 | Nodular | 9 | Yes | No | 6 |
| 179 | M | 69 | Acral | Yes | No | T2 | Done, positive | 50 | Acral lentiginous | 5 | No | No | 5 |
| 180 | F | 48 | Acral | No | No | T1 | Not done | 0 | Superficial spreading | 2 | No | No | 2 |
| 181 | M | 35 | Acral | No | No | T1 | Done, negative | 0 | Acral lentiginous | 15 | No | No | 15 |
| 182 | M | 74 | Acral | Yes | No | T3 | Done, negative | 4 | Acral lentiginous | 13 | No | No | 13 |
| 183 | F | 54 | Acral | Yes | No | T3 | Done, positive | 3 | Acral lentiginous | 18 | No | Yes | 16 |
| 184 | M | 54 | Acral | Yes | No | T1 | Done, negative | 3 | Acral lentiginous | 14 | No | No | 14 |
| 185 | M | 59 | Acral | No | No | T1 | Done, negative | 0 | Acral lentiginous | 12 | No | No | 12 |
| 186 | M | 77 | Acral | No | No | T3 | Done, negative | 1 | Acral lentiginous | 11 | No | No | 11 |
| 187 | M | 33 | Acral | No | No | T1 | Done, negative | 4 | Acral lentiginous | 10 | No | No | 10 |
| 188 | M | 51 | Acral | Yes | No | T4 | Done, negative | 37 | Nodular | 14 | Yes | Yes | 10 |
| 189 | F | 67 | Trunk | Yes | No | T3 | Done, negative | 17 | Nodular | 14 | No | Yes | 10 |
| 190 | M | 63 | Acral | No | No | T4 | Done, positive | 12 | Nodular | 14 | No | Yes | 3 |
| 191 | F | 58 | Upper/lower extremity | No | No | T1 | Done, negative | 2 | Superficial spreading | 12 | No | No | 12 |
| 192 | F | 44 | Upper/lower extremity | No | No | T1 | Not done | 1 | Superficial spreading | 6 | No | No | 6 |
| 193 | F | 35 | Upper/lower extremity | No | No | T1 | Done, negative | 2 | Nodular | 6 | No | No | 6 |
| 194 | M | 31 | Upper/lower extremity | Yes | No | T1 | Done, negative | 2 | Nodular | 10 | No | No | 10 |
| 195 | M | 54 | Acral | Yes | No | T4 | Done, negative | 2 | Desmoplastic | 6 | No | No | 6 |
| 196 | M | 54 | Acral | No | No | T1 | Done, negative | 2 | Acral lentiginous | 11 | No | No | 11 |
| 197 | F | 56 | Acral | No | NA | T1 | Done, negative | NA | Acral lentiginous | 2 | No | No | 2 |
| 198 | M | 58 | Acral | No | No | T1 | Not done | 5 | Acral lentiginous | 8 | No | No | 8 |
| 199 | F | 79 | Acral | Yes | No | T2 | Done, negative | 4 | Acral lentiginous | 5 | No | No | 5 |
| 200 | F | 52 | Head/neck | No | No | T2 | Not done | 11 | Superficial spreading | 10 | Yes | No | 5 |
| 201 | M | 65 | Acral | No | No | T4 | Done, negative | 18 | Acral lentiginous | 9 | Yes | Yes | 4 |
| 202 | M | 27 | Trunk | No | No | T3 | Not done | NA | Superficial spreading | 43 | No | Yes | 10 |

NA, not accessed
